# Impact of Secondhand Smoke on the Biomineralization of Dental Enamel in Rats

**DOI:** 10.1007/s00223-026-01542-6

**Published:** 2026-05-08

**Authors:** Juliana de Lima Gonçalves, Alice Corrêa Silva-Sousa, Manoel Damião de Sousa-Neto, Fabrício Kitazono de Carvalho, Alexandra Mussolino de Queiroz, Bernhard Ganss, Francisco Wanderley Garcia de Paula-Silva

**Affiliations:** 1 Department of Pediatric Dentistry, Ribeirão Preto School of Dentistry, University of São Paulo, Ribeirão Preto, São Paulo Brazil; 2 Department of Restorative Dentistry, Ribeirão Preto School of Dentistry, University of São Paulo, Ribeirão Preto, São Paulo Brazil; 3 Faculty of Dentistry, University of Toronto, Toronto, ON Canada

**Keywords:** Secondhand smoke, Dental enamel defect, Adverse events, Early childhood development

## Abstract

This study aims to investigate the qualitative and quantitative morphological changes in dental enamel resulting from SHS exposure during early childhood, utilizing an animal model. Wistar rat offspring were divided into a control group (*n* = 15) and an SHS exposure group (*n* = 19). The experimental group was subjected to cigarette smoke twice daily for 5 min, from postnatal day 2 to day 28. Throughout the experimental period, growth was monitored by measuring both weight and body length. Dental enamel was assessed through photographic analysis, micro-computed tomography (micro-CT), scanning electron microscopy (SEM), energy-dispersive X-ray spectroscopy (EDS), and microhardness testing. Mandibles were processed, followed by hematoxylin and eosin staining. Data analysis was conducted using Student’s t-tests (⍺= 5%). There was a significant reduction in weight gain in the SHS-exposed group compared to controls (*p <* 0.0001). Macroscopic examination revealed no structural differences in the incisors or molars of SHS-exposed offspring relative to controls. SEM analysis demonstrated increased spacing between superficial enamel prisms was noted in the SHS group. In terms of chemical composition, significant decreases in phosphorus content (*p* = 0.033) and increases in carbon content (*p =* 0.017) were detected in the molars of the SHS group, with no significant differences found in calcium, oxygen, or the calcium-to-phosphorus ratio. Micro-CT analysis showed no changes in enamel volume or microhardness, and no significant differences were detected in ameloblast morphology. In conclusion, while SHS exposure did not induce macrostructural changes in enamel, it was associated with reduced phosphorus levels, increased carbon levels, and greater spacing between superficial enamel prisms leading to a mild disturbance in dental mineralization.

## Introduction

According to the World Health Organization, it is estimated that at least two billion people globally are exposed to second-hand smoke (SHS), resulting in approximately 600,000 fatalities [1, 2]. The adverse effects of SHS exposure are comparable to those experienced by active smokers, particularly in vulnerable populations, such as pregnant women and children [3, 4]. Exposure to tobacco smoke during pregnancy and early childhood has been linked to various negative outcomes, including premature birth, low birth weight, neurocognitive impairment, oral clefts, as well as cardiovascular and respiratory diseases [4–7]. Several studies indicate that SHS exposure can also adversely affect tooth development, given that odontogenesis commences around the gestation period [8–10].

Early investigations into the relationship between cigarette smoke exposure and dental alterations reported morphological differences in dental crowns [11, 12]. Also, children of smoking mothers were 4.95 times more likely to exhibit teeth with shortened dental root compared to children not exposed to maternal smoking [10]. Furthermore, SHS has been identified as a risk factor for dental caries in children [13]. Studies utilizing animal models have demonstrated that maternal smoking can lead to arrested morphological development and impaired mineralization of teeth [9]. A prospective cohort study performed with mothers of twins demonstrated a stronger association between maternal smoking and the occurrence of hypomineralized second primary molars, demonstrating that the components of cigarette smoke can impair ameloblasts activity during enamel formation [14].

Amelogenesis is the process of dental enamel development and involves a complex regulated sequence of cellular and molecular events such as calcium (Ca²⁺) and phosphate (PO_4_³⁻) incorporation into enamel matrix, and protein removal [15, 16]. Disruptions during this process could potentially result in dental enamel defects (DEDs) [16, 17]. During the secretion stage of amelogenesis, ameloblasts are responsible for releasing proteins such as amelogenin, ameloblastin, and enamelin. These proteins position themselves around hydroxyapatite crystals to form a scaffold that guides the growth of enamel prisms [15]. Subsequently, during the mineralization stage, ameloblasts release enzymes such as kallikrein 4 (KLK4) and matrix metalloproteinase 20 (MMP20), which remove organic content from the dental enamel matrix and facilitate the liberation of significant amounts of Ca²⁺ and PO_4_³⁻ that are incorporated into hydroxyapatite crystals. Any failures during these processes can lead to enamel characterized by lower mineral content, diminished surface resistance, and increased porosity [15–17].

Studies investigating the prevalence of dental enamel defects (DEDs) indicate considerable variability influenced by several factors, including the type of defect, geographic region, diagnostic methodology, age, and type of dentition [18, 19]. DEDs exhibit a multifactorial etiology involving genetic, systemic, and environmental factors that can act during the pre-natal, peri-natal, and post-natal periods, with a predominant influence during early childhood [19].

The adverse effects of exposure to cigarette smoke during the early years of life on child development and oral health outcomes are well documented [11–13] however, research concerning the specific impacts of chronic second-hand smoke (cSHS) on dental enamel remains insufficiently established. Consequently, this study aims to investigate the qualitative and quantitative morphological changes in dental enamel associated with SHS exposure during early childhood, utilizing an animal model.

## Materials and Methods

### Animals

Following approval from the Animal Ethics Committee (0062/2023), the animals were maintained in accordance with the Brazilian Guidelines for the Care and Use of Animals in Teaching or Scientific Research Activities, as regulated by the National Council for the Control of Animal Experimentation (Law 11.794/2008). All experimental procedures adhered to the ARRIVE guidelines.

For this study, four dams were used, resulting in a total of 34 (21 male and 13 female) alive newborn Wistar rats (*Rattus norvegicus albinus*), each weighing approximately 20 g. Two damns were allocated to each group (control = 2 and experimental = 2). For control group, the first dam delivered five pups (five males and one female) that were euthanized in 14 day. The second dam from control group delivered nine pups (five male and four female) that were used for the analysis of 28 day. In experimental group, the first dam delivered eight pups (six male and two female), which were euthanized at 14 days of age, while the second dam delivered 11 pups (five male and six female), which were euthanized at 28 days of age. The animals were housed in an Animal Facility at the School of Dentistry of Ribeirão Preto, University of São Paulo. The rats were kept in polypropylene cages equipped with stainless steel lids, maintained under controlled temperature conditions (22 °C ± 10%) and a 12:12 h light-dark cycle. They had access to a standard laboratory diet and were provided with ad libitum access to filtered water. All pups were kept with their respective mothers until they reached 21 days of age, at which point sexing was performed, and the animals were separated by sex.

### Experimentation

The animals in the second-hand smoke (SHS) group were separated from their mothers and placed in a specially designed transparent acrylic device (45 × 25 × 20 cm³; 22500 cm³) that allowed for controlled exposure to both cigarette smoke and ambient air. Exposure sessions occurred twice daily, with an interval of 12 h, for 5 min each, utilizing five Marlboro® cigarettes (Philip Morris, Richmond, VA, USA) per session, starting on postnatal day two and continuing until day 28 [20]. This exposure protocol was adapted from Arnez et al. [20], with the duration of each session increased from three to five minutes. After each exposure, the pups were returned to their mothers. In contrast, control animals remained in ambient air and were not exposed to cigarette smoke.

For euthanasia, the animals were first anesthetized with a combination of 10% ketamine hydrochloride (100 mg/kg; Química Farmacêutica Nacional Union Agener S/A, Embu-Guacu, SP, Brazil) and 2% xylazine (7.5 mg/kg; Dopaser, Labs Calier S/A, Barcelona, Spain). Following anesthesia, the rats were placed in a CO₂ chamber for euthanasia. Subsequently, the lower and upper incisors, along with tissues containing bone and teeth, were collected for further analysis.

On the 14th day post-birth, euthanasia was performed on the control group (*n* = 6) and the SHS group (*n* = 8) in order to evaluate the amelogenesis of the first molars. A second euthanasia was conducted on day 28 for the control group (*n* = 9) and the experimental group (*n* = 11), from which erupted first molars and incisors were collected.

### Evaluation of Body Weight and Length

Throughout the experimental period, the body weight of the offspring in both groups was closely monitored. Weights and measurements of the experimental (SHS) and control groups were recorded on the 1 st, 7th, 14th, 21 st, and 28th days of the study. Tracking changes in body weight and length served as indicators of the physical development of the animals, enabling the detection of any potential differences between the control and SHS groups.

### Macrophotography

To analyze the presence of clinically visible changes in enamel and facilitate subsequent comparisons between the experimental and control groups, photographs of the incisors and molars of the animals were captured. For macrophotography of the incisors, images were taken following euthanasia. A Canon EOS Rebel T2i camera (Canon, Tokyo, Japan) equipped with a 100 mm f/2.8 macro lens (Canon, Tokyo, Japan) was utilized, with the camera settings adjusted to ISO 200, f/32, and a shutter speed of 3 s, using a polarized light filter. Photographs were taken of the labial aspects of both the upper and lower incisors.

For the molars, mandibles containing the molars were collected and stored in distilled water. After air-drying, photographs were taken of the labial and occlusal surfaces using a stereo microscope (Amscope, Irvine, California, USA).

### Micro-computed Tomography

A microtomographic analysis was conducted on the incisors and lower molars of the animals to evaluate the enamel layer. After undergoing a thorough cleaning and drying process, the incisors and molars were individualy placed into microtubes positioned perpendicular to the radiation source to facilitate accurate imaging. The samples were then scanned using a SkyScan 1174 micro-CT scanner (Bruker, Kontich, Belgium). The scanning was performed using 50 kV and 800 µA power, with an isotropic resolution of 17.3 μm, a 360° rotation around the vertical axis, and a rotation step of 0.6°. The X-ray beam was filtered using a 0.5 mm thick aluminum filter.

Subsequent to scanning, the axial sections were reconstructed from the angular projection images using the modified Feldkamp cone-beam reconstruction algorithm, implemented in the NRecon software (v.1.7.4.2, Bruker-microCT). The reconstructed images were then analyzed using 3D Slicer software (Wayne Rasband, National Institutes of Health, USA). Sagittal section images were acquired with the incisal edge serving as a reference point, extending 6 mm toward the cervical region, terminating at a defined yellow line. The total volume of the enamel layer on both incisors and molars was calculated after segmenting the enamel layer using ITK-SNAP software [21].

### Knoop Microhardness Test

Microhardness testing was conducted on the subsurface enamel layer of both incisors and molars. For the incisors, six right maxillary tooth were used for each group. The tooth were embedded in epoxy resin, and subsequently ground and polished using 200-, 600-, and 1200-grit papers to expose the full enamel layer in the sagittal plane. Indentations were made in the incisal and cervical regions of the incisors. The first series of there indentations, with a distance of 20 μm between them were performed with a distance of 200 μm from the incisal edge. Then, a distance of 500 μm from the first series of indentation was used for the second series, and an additional 500 μm for the third series, each consisting of three indentations [22].

For the molars, six right maxillary first molars of each group were selected for analysis. The same sample preparation protocol from the incisors was performed. A Indentations were performed on the lingual surface at the mesial cusp. For the first series of indentations, a distance of 200 μm from the beginning of the occlusal surface was used with three indentations spaced 20 μm apart. The second and third series were also performed with three indentations each, maintaining a distance of 20 μm between indentations and 200 μm between each series, considering the total length of the mesial surface of the molars. The average hardness of each tooth was then calculated to assess changes in enamel hardness, which may indicate alterations in enamel composition due to smoke exposure. A microdurometer ((Shimadzu–HMV-2, Kyoto, Japan) equipped with a Knoop diamond tip, applying a load of 10 gf for 10 s, was utilized for this measurement.

### Energy Dispersive X-Ray Analysis (EDS) and Scanning Electron Microscopy (SEM)

For the quantification of mineral content and elemental representation, energy-dispersive X-ray spectroscopy (EDS) analysis was performed using a JSM-6610LV scanning electron microscope (JEOL, Tokyo, Japan) equipped with INCA software (Oxford Instruments, Abingdon, United Kingdom). Analyses were conducted at low vacuum mode at 20 kV, with a working distance of 12 mm and an acquisition time of 50 s. Four left mandibular incisors from each animal/and group were sonicated in distilled water for 1 min, and then air drying for 24 h at room temperature. Measurements were obtained using point analysis in three regions: incisal, transition region (yellow line used as reference), and cervical. Teeth were positioned perpendicular to the incident beam, and three point analyses were performed, with an average distance of 5 mm between each measurement.

For the molars, four left mandibular first molars from each group were prepared following the same protocol as the incisors. Samples were positioned so that the electron beam was perpendicular to the cuspal surfaces of the molars. Using the point analysis tool in the software, one measurement was obtained from each cusp. Elemental content was calculated as the relative weight% of the total element composition (100%). The primary elements analyzed included calcium (Ca), phosphorus (P), oxygen (O), and carbon (C), and the calcium-to-phosphorus (Ca/P) ratio was subsequently calculated.

For scanning electron microscopy (SEM), the same incisors used in EDS was used to evaluate the structure of enamel prisms and identify potential morphological changes at the microscopic level. The four left lower incisors from each group were embedded in self-curing acrylic resin inside circular tubes and cross-sectioned at the mid-portion, beginning at the yellow part. The samples were then subjected to surface conditioning with 37% phosphoric acid for 40 s, followed by thorough washing with distilled water, sonicated for one minute and air drying for 24 h at room temperature.

Imaging was performed using a Hitachi FlexSEM 1000 (Hitachi, Ibaraki, Japan) scanning electron microscope at an accelerating voltage of 10 kV and a working distance of approximately 10 mm, using a secondary electron detector. The microscope was operated under low-vacuum conditions, allowing the analysis of non-conductive samples without metal coating. Images of the enamel layer were acquired at magnifications of 150×, 750×, and 3000×.

### Histological Processing and Hematoxylin and Eosin Staining

The mandibles of the animals were collected and fixed in 10% formaldehyde for 24 h. Following fixation, the mandibles were washed with water for 4 h and then demineralized in a 5% solution of ethylenediaminetetraacetic acid (EDTA; pH 7.4) for a period of 40 days. After demineralization, the samples were embedded in paraffin, and blocks containing the incisors and molars were cut into sagittal sections. Subsequently, hematoxylin and eosin (HE) staining was performed on the slides prepared from the animals at the 14th and 28th days of age.

#### Statistical Analysis

The results obtained were analyzed using GraphPad Prism 8.0 software (Prism 8, Chicago, IL, USA). Developmental data, including weight and length, were subjected to analysis of variance (ANOVA), followed by Tukey’s post-hoc test for multiple comparisons. Data from micro-CT volume measurements, microhardness testing, and energy-dispersive spectroscopy (EDS) were first assessed for normality, and subsequently analyzed using the parametric Student’s t-test. A significance level of 5% was established for all analyses.

## Results

### Evaluation of Body Weight and Length

It was observed that animals that were exposed to tobacco smoke had a lower mean of weight at 28th day of life (76.82 g ± 4.60) when compared to control group (86 g ± 3.16) (*p* < 0.0001) (Table 1). Meanwhile, the length evaluation had no differences between SHS (11.79 cm ± 0.4) and control (12.12 cm ± 0.5) (*p =* 0.472) (Table 2).

**Table 1:** Body weight (g) of rat pups over the experimental period

Group		Day 1	Day 7	Day 14	Day 21	Day 28
Control	Litter 1 (*n* = 6)	6.58 ± 0.48	20 ± 1.78	37 ± 2.00	-	-
	Litter 2 (*n* = 9)	6.6 ± 1.27	15.94 ± 1.43	31.62 ± 2.29	50.22 ± 2.43	86 ± 3.16
SHS	Litter 3 (*n* = 8)	7.13 ± 0.32	17.49 ± 0.47	33 ± 1.09	-	-
	Litter 4 (*n* = 11)	6.72 ± 0.37	14.58 ± 0.59	27.38 ± 0.65	42.82 ± 1.60*	76.82 ± 4.60*

Values are expressed as mean ± standard deviation per litter. Pups were evaluated at days 1, 7, 14, 21, and 28. Animals from Litter 1 in both control and SHS groups were euthanized at day 14; therefore, data for these groups are presented up to this time point. * indicates p value difference between groups

**Table 2:** Body length (cm) of rat pups over the experimental period

Group		Day 7	Day 14	Day 21	Day 28
Control	Litter 1 (*n* = 6)	7.05 ± 0.26	9.85 ± 0.53	-	-
	Litter 2 (*n* = 9)	6.69 ± 0.69	8.50 ± 0.28	9.99 ± 0.45	12.12 ± 0.56
SHS	Litter 3 (*n* = 8)	6.98 ± 0.28	8.57 ± 0.19	-	-
	Litter 4 (*n* = 11)	6.45 ± 0.49	8.57 ± 0.27	10.15 ± 0.45	11.79 ± 0.44

Values are expressed as mean ± standard deviation per litter. Pups were evaluated at days 7, 14, 21, and 28. Animals from Litter 1 in both control and SHS groups were euthanized at day 14; therefore, data for these groups are presented up to this time point. * indicates *p* value difference between groups

### Macrophotography

**Fig. 1:**
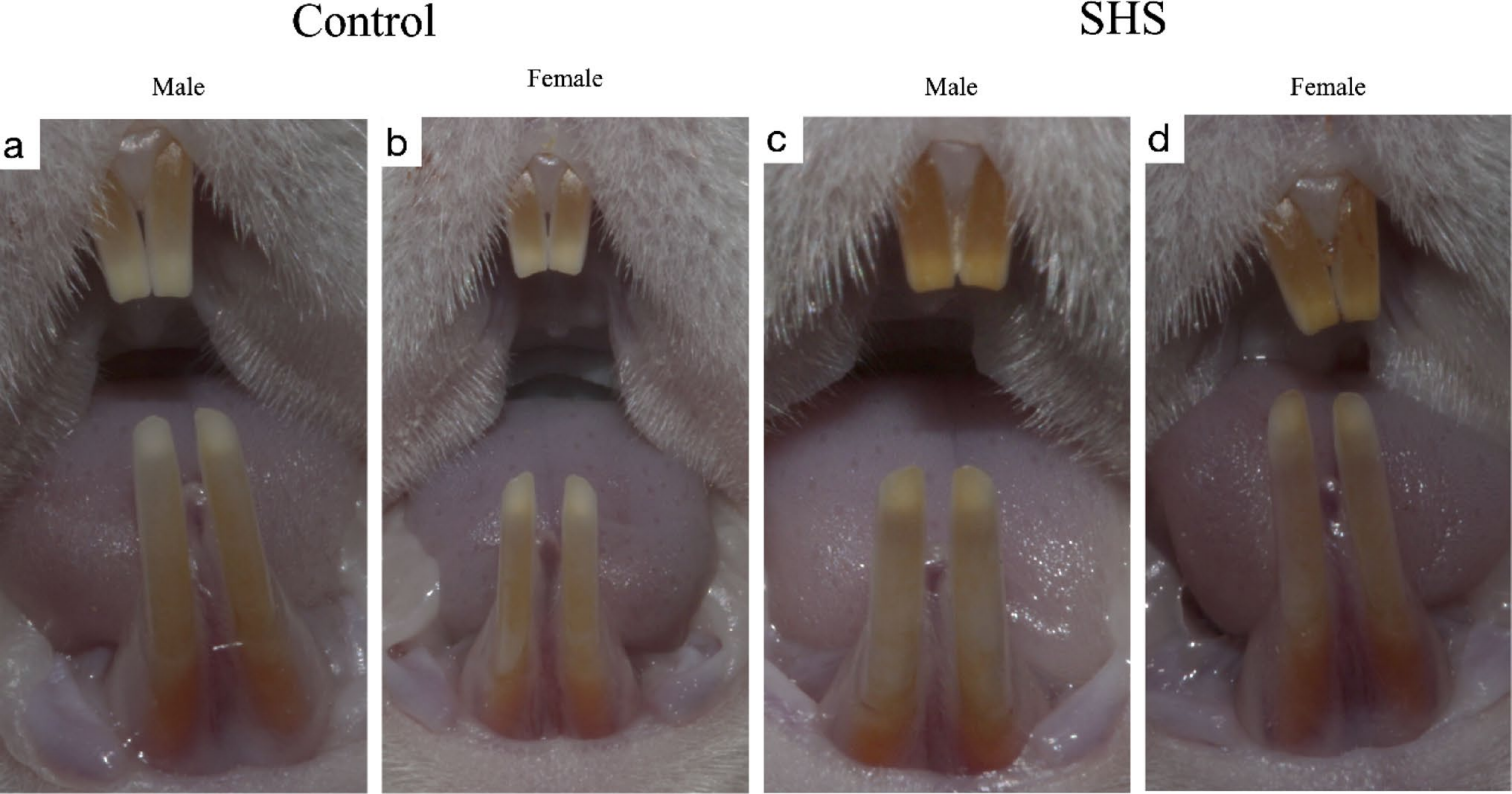
Macrophotography of the lower and upper incisors from the control (**a**, **b**) and SHS groups (**c**, **d**) was performed using a polarized light filter. The images reveal that the animals in the tobacco smoke exposure group displayed yellowish teeth compared to those in the control group. However, no qualitative or quantitative dental enamel defects were identified, and there were no differences observed between sexes.

**Fig. 2:**
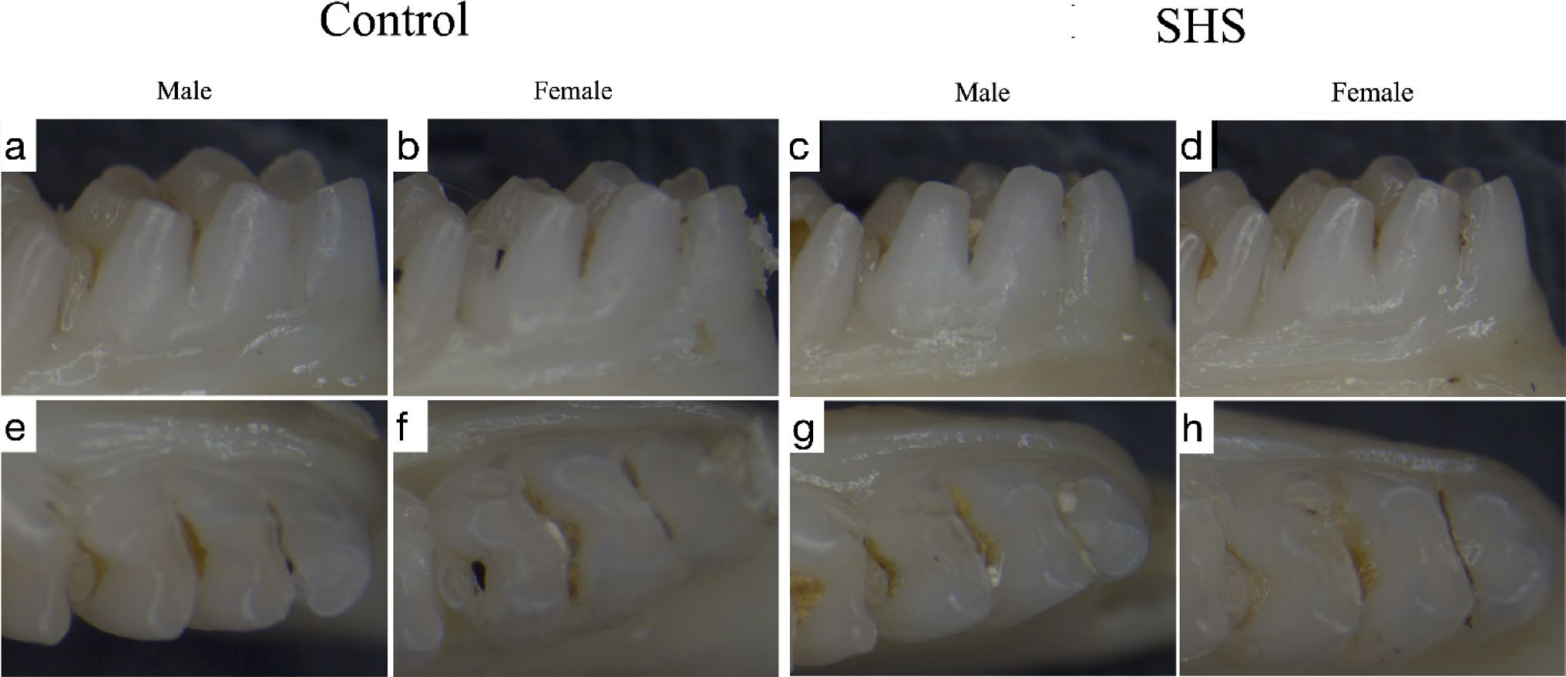
Aspects of the labial surface of the first molars from the control (**a**, **b**) and SHS (**c**, **d**) groups are presented, along with the occlusal surface of the control (**e**, **f**) and SHS (**g**, **h**) groups. No differences in color, structural integrity, or opacities were observed between the groups or between sexes.

No visual differences were observed between the groups; however, the animals in the SHS group exhibited yellowish incisors compared to the control animals (Fig. 1). Additionally, no qualitative or quantitative dental enamel defects were identified, and there were no discernible differences in the incisors or molars based on sex (Fig. 2).

### MicroCt

**Fig. 3:**
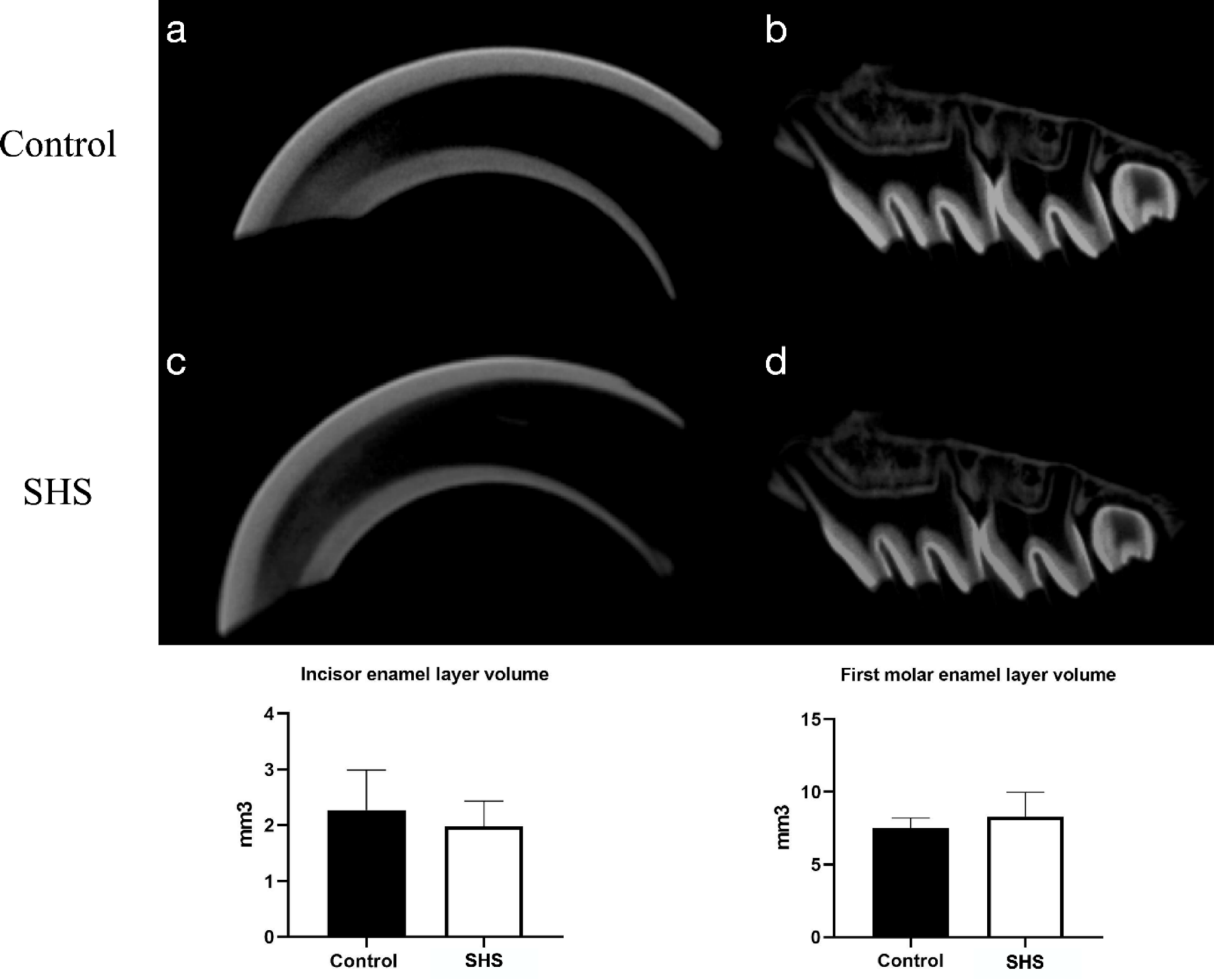
Micro-CT analysis of the enamel of upper incisors and molars from the control and SHS groups is presented in sagittal sections. Images depict the incisors (**a**, **c**) and the mandibles containing molars (**b**, **d**) for both groups.

The volumetric analysis of the enamel layer revealed no statistically significant differences between the upper incisors of the control group (1.98 mm³ ± 0.26) and the SHS group (2.27 mm³ ± 0.49) (*p* = 0.581). Additionally, analyses conducted on the enamel layer of the maxillary first molar also showed no differences between the control group (7.47 mm³ ± 0.82) and the SHS group (8.29 mm³ ± 1.05) (*p* = 0.481) (Fig. 3).

### Energy Dispersive X-Ray Analysis (EDS) and Microhardness

Energy-dispersive spectroscopy (EDS) was performed on the incisors and molars of animals at 28 days of age. The analysis revealed no significant difference in calcium content between the two groups. However, the SHS group exhibited a decrease in phosphorus content (*p* = 0.033) and an increase in carbon content (*p* = 0.017) when compared to the control group. The calcium-to-phosphorus (Ca/P) ratio showed no significant differences between the groups. Additionally, the analysis of oxygen content indicated no differences in the quantities of these element between the groups (Table 3).

**Table 3:** Energy dispersive X-ray analysis results from the incisors and molars

Group		Ca	*P*	O	C	Ca/*P*
Control	Incisor	24.10 (± 0.68)	13.11 (± 0.04)	41.81 (± 0.28)	20.99 (± 0.28)	2.58 (± 0.47)
	Molar	31.12 (± 2.65)	18.48 (± 1.69)*	46.33 (± 2.28)	6.38 (± 2.28)*	1.82 (± 0.24)
SHS	Incisor	27.59 (± 2.84)	14.54 (± 0.94)	40.34 (± 0.86)	40.34 (± 0.86)	2.01 (± 0.12)
	Molar	25.76 (± 1.84)	13.48 (± 0.95)*	45.26 (± 2.04)	45.26 (± 2.04)*	2.09 (± 0.27)

The values express the average (± standard deviation). Ca: calcium; P: phosphorus; O: oxygen; C:carbon; Ca/P: calcium and phosphorus ratio. * indicates significant difference

Concerning the microhardness of the enamel surface, indentations were performed on the incisors and molars. The results indicated no significant differences between the groups in both the tip (*p* = 0.958) and cervical regions of the incisors (*p* = 0.18). Similarly, the labial surface of the molars showed no differences between the control and SHS groups (*p* = 0.559; Table 4).

**Table 4:** Microhardness values in KHN from incisors and molars for control and SHS groups

Group/tooth region	Incisor		Molar
	Tip	Cervical	Occlusal-cervical
Control	194 (± 49.9)	240 (± 30.7)	220 (± 83.8)
SHS	193 (± 40.5)	185 (± 59.3)	227 (± 33.7)

The values are the average (±standard deviation)

### Scanning Electron Microscopy (SEM)

**Fig. 4:**
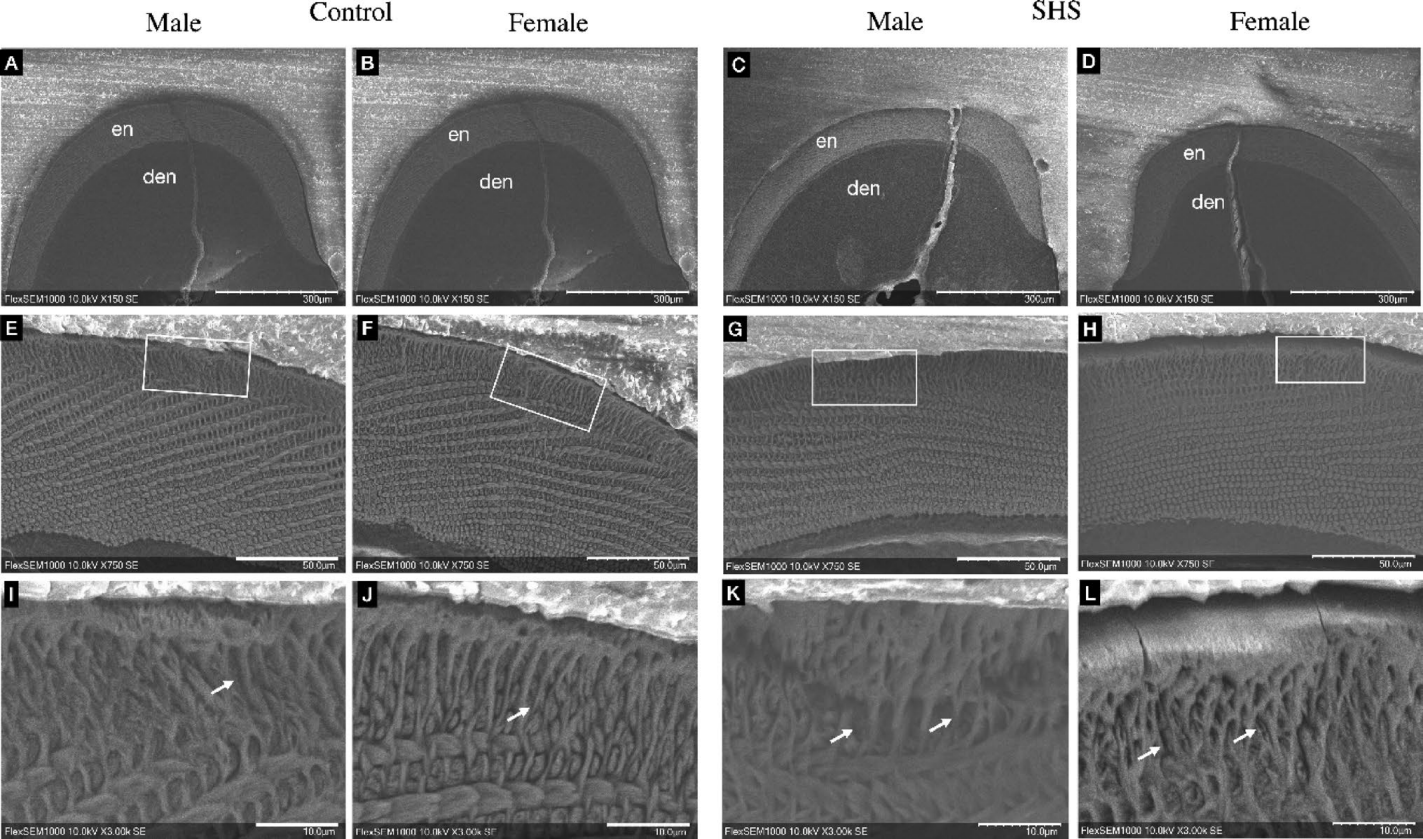
Cross-sectional SEM images of enamel from control and SHS groups in male and female animals. Images were obtained at different magnifications: (**a**, **b**, **c** and **d**, scale bar = 300 μm) 150x magnification, (**e**, **f**, **g**, and **h**, scale bar = 50 μm) 750x magnification, and (i, k, k and l, scale bar = 10 μm) 3000x magnification. At higher magnification, the SHS group exhibited increased spacing between enamel rods in the aprismatic region (white arrows). En: enamel; den: dentin.

After analyzing the ultrastructure of the enamel layer among the groups and between male and female animals, no differences were observed in the distribution and orientation of hydroxyapatite crystals within the prismatic region. Both the prismatic and interprismatic regions demonstrated similarities between the groups. However, in the outermost portion of the enamel layer, known as the aprismatic region - characterized by crystals lacking an orderly arrangement and prisms aligned parallel to one another, a greater spacing between prisms was observed in the smoke-exposed group compared to the control group (Fig. 4).

### Hematoxylin and Eosin Staining

**Fig. 5:**
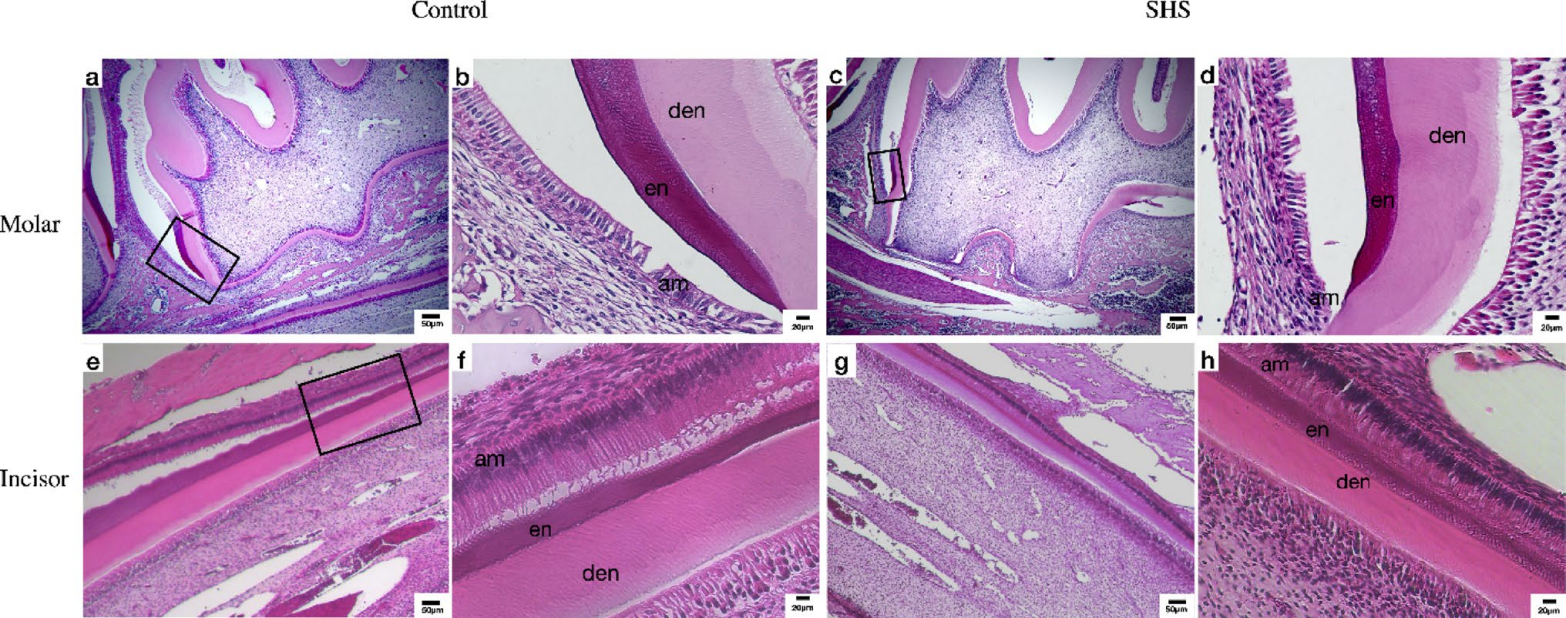
Hematoxylin and eosin staining of molars (**a** and **c**, scale bar = 50 μm; **b** and **d**; scale bar = 20 μm) and incisors (**e** and **g**, scale bar = 50 μm; **f** and **h**, scale bar = 20 μm) from control animals and from SHS animals. Am: ameloblasts; En: enamel; Den: dentin.

Hematoxylin and eosin staining enabled the evaluation of potential differences in the morphology of ameloblasts and their cellular structures, including Tomes’ process and nuclear cell polarization. The analysis was conducted on the molars of animals at postnatal day 14, revealing that enamel matrix mineralization was already at an advanced stage, as indicated by the presence of enamel space that was demineralized during histological processing. In the cervical regions of the dentinoenamel junction, ameloblasts in the matrix mineralization stage were still evident; however, no morphological differences were identified between the groups (Fig. 5).

For the incisors, the analysis was conducted on animals at postnatal day 28, as this tooth undergoes continuous development, allowing for the observation of all stages of amelogenesis along its axis. Both secretory and maturation-stage ameloblasts exhibited no morphological differences between the groups (Fig. 5).

## Discussion

Secondhand smoke (SHS) affects approximately 34.3% of the global population and is associated with health risks comparable to those observed in active smokers, including ischemic heart disease, chronic obstructive pulmonary disease, and lower respiratory infections [1]. During early childhood, SHS can impair physical development and is linked to an increased risk of various diseases, including respiratory illnesses, asthma, and obesity [1, 23].

The present study demonstrated that rodent offspring exposed to SHS had a lower average body weight compared to their non-exposed counterparts, although no differences were observed in average body length. Human studies have indicated that SHS exposure during pregnancy is associated with higher rates of preterm birth and low birth weight [7, 24]. The impact of SHS during the prenatal period can be attributed to the absorption of toxic components from tobacco smoke, which can cross the placental barrier and affect the fetus.

One significant compound in tobacco smoke is carbon monoxide, a molecule with a high affinity for hemoglobin. When carbon monoxide binds to hemoglobin, it forms carboxyhemoglobin, which reduces the blood’s oxygen-carrying capacity. This reduction can lead to decreased oxygen delivery to fetal tissues, potentially resulting in hypoxia and impaired fetal development [24, 25]. Although exposure to cigarette smoke is correlated with low birth weight, studies have shown that children exposed to tobacco smoke during both prenatal and postnatal periods are at a heightened risk of developing obesity later in life [24, 26, 27]. A study where nicotine was administered during the gestational and lactational periods in rats indicated that the offspring had increased body fat accumulation and a predisposition to diabetes mellitus. Although the underlying mechanisms remain partially understood, it has been hypothesized that nicotine may induce apoptosis of type B cells, which play a role in regulating the immune response. An increased rate of apoptosis in these cells has been associated with elevated oxidative stress levels, leading to a reduction in insulin concentrations in the bloodstream. Decreased insulin levels can result in glucose accumulation in the blood, potentially resulting in the onset of diabetes [28].

In addition to its effects on general child health, secondhand smoke (SHS) can also directly impact oral health, including tooth development and mineralization [13]. Studies have indicated a higher prevalence of dental caries associated with SHS, which may be attributed to nicotine’s ability to promote the proliferation of *Streptococcus mutans*, a bacteria implicated in the pathogenesis of dental caries [29–31]. Other consequences of SHS exposure include shortening of dental roots [10], defects in enamel mineralization [9] and hypomineralized second primary molars [14].

Enamel hypomineralization can occur due to disturbances during the mineralization stage of amelogenesis. During this stage, ameloblasts release enzymes that degrade the organic enamel matrix, while actively transporting calcium and phosphate ions that are incorporated into hydroxyapatite crystals [15–17]. The presence of opacities in the enamel is often the result of accumulated organic content, while hypomineralized regions are characterized by lower mineral content and density, reduced hardness, and disorganized enamel prisms [17, 32]. In the present study, no clinical evidence of opacities or loss of enamel structure was observed in the incisors or molars of animals exposed to cigarette smoke. Additionally, studies investigating potential etiological factors, such as antibiotic use and stress induction, also reported no clinically visible alterations on the enamel surface [33, 34].

In the elemental analysis, a lower incorporation of phosphorus and a higher content of carbon were observed in the molars of animals exposed to tobacco smoke. This reduced mineral incorporation in hydroxyapatite crystals may result in decreased hardness of the enamel layer and structural alterations within the hydroxyapatite crystals, thereby compromising mechanical properties such as strength and increasing susceptibility to fractures [32, 35]. The increased carbon content in hypomineralized teeth indicates a higher presence of organic material in dental enamel, further supporting the alterations in the chemical and mechanical properties associated with this type of defect [17, 35]. In the present study, while no changes in enamel hardness values were observed for the incisors and molars, scanning electron microscopy revealed increased spacing between the enamel prisms in the outermost layer. It is important to highlight that microhardness testing is not a very sensitive method to detect mild changes in the enamel structure, such as those observed in animals exposed to secondhand smoke.

Structural alterations in enamel prisms and a reduction in enamel hardness was reported in enamel of offspring from rodents whose mothers were exposed to mercury during pregnancy. Scanning electron microscopy revealed disorganized and structurally compromised enamel prisms in these animals, which may have contributed to a loss of mechanical strength [22]. Exposure to toxic substances, such as mercury, during pregnancy and early childhood can adversely affect child development, as these substances are capable of crossing the placental barrier and reaching the fetus, potentially altering critical cellular signaling pathways [22, 36, 37]. Offspring of pregnant rats exposed to cigarette smoke presented impaired enamel biomineralization characterized by a reduced calcium-to-phosphorus ratio - an indicator of lower enamel mineral density. Furthermore, micro-CT analysis revealed decreases in both the thickness and mineral density of the molar enamel layer in animals exposed to cigarette smoke [9]. In the present study, while the total enamel layer volume of molars and incisors was quantified, no differences were observed between the groups. The reduction in enamel volume reported previously may be associated with the effects that toxic substances, such as nicotine, have on ameloblast activity during amelogenesis [9]. The dosage used in the present study was derived from prior research by Arnez et al. [20], which investigated the effects on mineralization signaling in palatal sutures. In contrast, the study by Dong et al. [9] examined the effects of passive smoking on enamel mineralization and employed a higher exposure dose; animals were subjected to cigarette smoke for two hours, demonstrating significant effects on enamel formation and mineralization. Thus, the administered dose of toxic substances is a critical factor in determining the effects on cellular signaling pathways, the development and formation of various structures, and the extent of damage.

Histologically, the results show that ameloblasts did not present morphological differences between groups, indicating that the dose of cigarette smoke exposure was not sufficient to impact enamel formation at the cellular level. Although SHS did not cause cellular-level alterations, studies that have investigated the effects of systemic nicotine administration during the gestational period in animals show that there is an impact on tooth germ formation [39, 40]. Systemic nicotine administered to animals during the prenatal period caused a delay in the stages of tooth germ formation in the treated offspring. In addition, it has also been observed that the administration of this substance causes morphological alterations in ameloblasts and odontoblasts, with changes in nuclear polarization and orientation. These effects have been attributed to nicotine induced interference with the metabolic activity of dental papilla cells, leading to impaired cell differentiation and delayed tooth development [38]. It has also been reported that nicotine has the ability to inhibit mineralization in human dental pulp cells [40]. When evaluating mineralization parameters in odontoblasts, it was observed that nicotine administration in these cells resulted in reducing alkaline phosphatase activity and mineralized nodule formation compared to the control group. Thus, it suggests that nicotine interferes with mineralizing cells, potentially impacting the formation of mineralized tissues [39].

In addition to direct inhalation of cigarette smoke, another route of exposure during early childhood is through breast milk [40]. Women who smoke or are exposed to passive smoking during lactation absorb cigarette toxins that are subsequently transferred to the infant, leading to increased nicotine levels in the child’s system and potentially exacerbating the harmful effects of the substance [40, 41].

## Conclusion

Secondhand smoke (SHS) exposure in rodent offspring results in a mild disturbance in dental mineralization, causing a reduction of phosphorus content, an increase of carbon in the dental enamel, and alterations in the conformation of enamel prisms, leading to increased spacing. However, the cigarette smoke exposure protocol utilized in the present study did not induce any clinical changes on the enamel surface, nor did it affect the volume or mechanical properties of the enamel layer.

## Data Availability

All data used in this article are available from the corresponding author upon reasonable request.
